# Urinary Proteome Analysis of Global Cerebral Ischemia–Reperfusion Injury Rat Model via Data-Independent Acquisition and Parallel Reaction Monitoring Proteomics

**DOI:** 10.1007/s12031-022-02055-1

**Published:** 2022-08-03

**Authors:** Xiaopeng Sun, Qiujie Li, Jiajia Wang, Yuan Ma, Mingshan Wang, Weiwei Qin

**Affiliations:** 1 Department of Anesthesiology, Qingdao Municipal Hospital, Qingdao University, Qingdao, 266071 China; 2 Department of Anesthesiology, Qingdao Municipal Hospital, Dalian Medical University, Dalian, 116044 China

**Keywords:** Urine proteome, Biomarker, Cerebral ischemia–reperfusion injury, Animal model, Data-independent acquisition, Parallel reaction monitoring

## Abstract

Cerebral ischemia–reperfusion (I/R) injury is the leading cause of death in severe hypotension caused by cardiac arrest, drowning, and excessive blood loss. Urine can sensitively reflect pathophysiological changes in the brain even at an early stage. In this study, a rat model of global cerebral I/R injury was established via Pulsinelli’s four-vessel occlusion (4-VO) method. Overall, 164 urinary proteins significantly changed in the 4-VO rat urine samples compared to the control samples by data-independent acquisition (DIA) proteomics technique (1.5-fold change, *p* < 0.05). Gene Ontology annotation showed that the acute-phase response, the ERK1 and ERK2 cascade, endopeptidase activity, blood coagulation, and angiogenesis were overrepresented. After parallel reaction monitoring (PRM) validation, 15 differential proteins having human orthologs were verified as the potential urinary markers associated with cerebral I/R injury. Of these potential biomarkers, 8 proteins were reported to be closely associated with cerebral I/R injury. Nine differential proteins changed even when there were no clinical manifestations or histopathological cerebral damage, including FGG, COMP, TFF2, HG2A, KNG1, CATZ, PTGDS, PRVA, and HEPC. These 9 proteins are potential biomarkers for early screening of cerebral I/R injury to prevent the development of cerebral injury. KNG1, CATZ, PTGDS, PRVA, and HEPC showed an overall trend of upregulation or downregulation at 12 and 48 h after I/R injury, reflecting the progression of cerebral I/R injury. These 5 proteins may serve as potential biomarkers for prognostic evaluation of cerebral I/R injury. These findings provide important clues to inform the monitoring of cerebral I/R injury and further the current understanding of its molecular biological mechanisms.

**Supplementary Information:**

The online version contains supplementary material available at 10.1007/s12031-022-02055-1.

## Introduction

Global cerebral ischemia–reperfusion (I/R) injury is the leading cause of death in severe hypotension caused by cardiac arrest, drowning, and excessive blood loss (Benjamin et al. 2017). Because the brain is very sensitive to hypoxia, the brain tissue sustains irreversible damage 4 to 6 min after the loss of circulation, especially in highly metabolically active regions such as the hippocampus, thalamus, cerebral cortex, and striatum (Sanganalmath et al. 2017). Global cerebral I/R injury is closely associated with neurological dysfunction, from mild cognitive impairment to a minimally conscious state or even a persistent vegetative state (Bajan 2015). According to reports, up to 80% of survivors are in a vegetative state, and patients who recover well often have serious psychological complications such as depression, anxiety, and posttraumatic stress disorder, which seriously affect their quality of life (Shao et al. 2014). To date, the diagnosis and evaluation of global cerebral I/R injury have mainly relied on clinical symptoms, neuroimaging, electrophysiology, and biochemical examination of blood or cerebrospinal fluid. However, hemodynamic instability, sedation, and hypothermia are common in these patients, limiting the application of these tests (Nguyen et al. 2018). The main clinical treatment methods include supportive therapy, symptomatic treatment, mild hypothermia, and hyperbaric oxygen therapy. There is still a lack of specific, effective neuroprotective strategies and drugs. Therefore, early and accurate assessment of the degree of brain injury and prognosis is critical for diagnosis and treatment.

Currently, blood and cerebrospinal fluid are the main sources of samples for the study of brain injury biomarkers. Although many candidate protein biomarkers have been reported in blood and cerebrospinal fluid, they have not been further applied (Rech et al. 2006; Al-Mufti et al. 2017). There are several drawbacks to focusing on these fluids: (1) an invasive sampling process is necessary to obtain blood or cerebrospinal fluid, and the latter is especially difficult to obtain. (2) Blood and cerebrospinal fluid are important components of the internal environment; therefore, they are strictly controlled by homeostatic mechanisms to maintain the relative stability of their components. When a certain change is introduced into the internal environment, the body will reduce this change as much as possible and eliminate it through various mechanisms to keep its composition relatively stable. Therefore, changes in biomarkers in blood and cerebrospinal fluid are not sufficiently sensitive. (3) Due to technical limitations, the sample size and the extent of protein identification in previous studies were relatively small, and the conclusions have not been extensively validated. Thus, simple, non-invasive, and sensitive biomarkers are needed.

According to the largest human urine proteome database including 6085 sequences of protein information, some of these proteins have been reported to be enriched in 32 tissues and organs, such as the brain, gastrointestinal tract, and kidneys (MacLean et al. 2010). Among these proteins, 1956 that were enriched in brain tissue were identified in urine; this number ranked first among the 32 tissues and organs evaluated. Several studies have shown that urine can reflect the pathophysiology of some neurological diseases, such as Alzheimer’s disease, Parkinson’s disease, multiple sclerosis, and neuroendocrine tumors (An and Gao 2015). In addition, in a glioblastoma rat model and a Walker 256 lateral ventricle inoculation rat model, the urine proteome changed significantly before clinical symptoms and brain histopathological changes appeared (Zhang et al. 2019; Ni et al. 2018). Overall, urine is a good source of specimens for the study of brain injury markers, and it can provide a sensitive reflection of brain pathophysiology at an early stage.

**Fig. 1:**
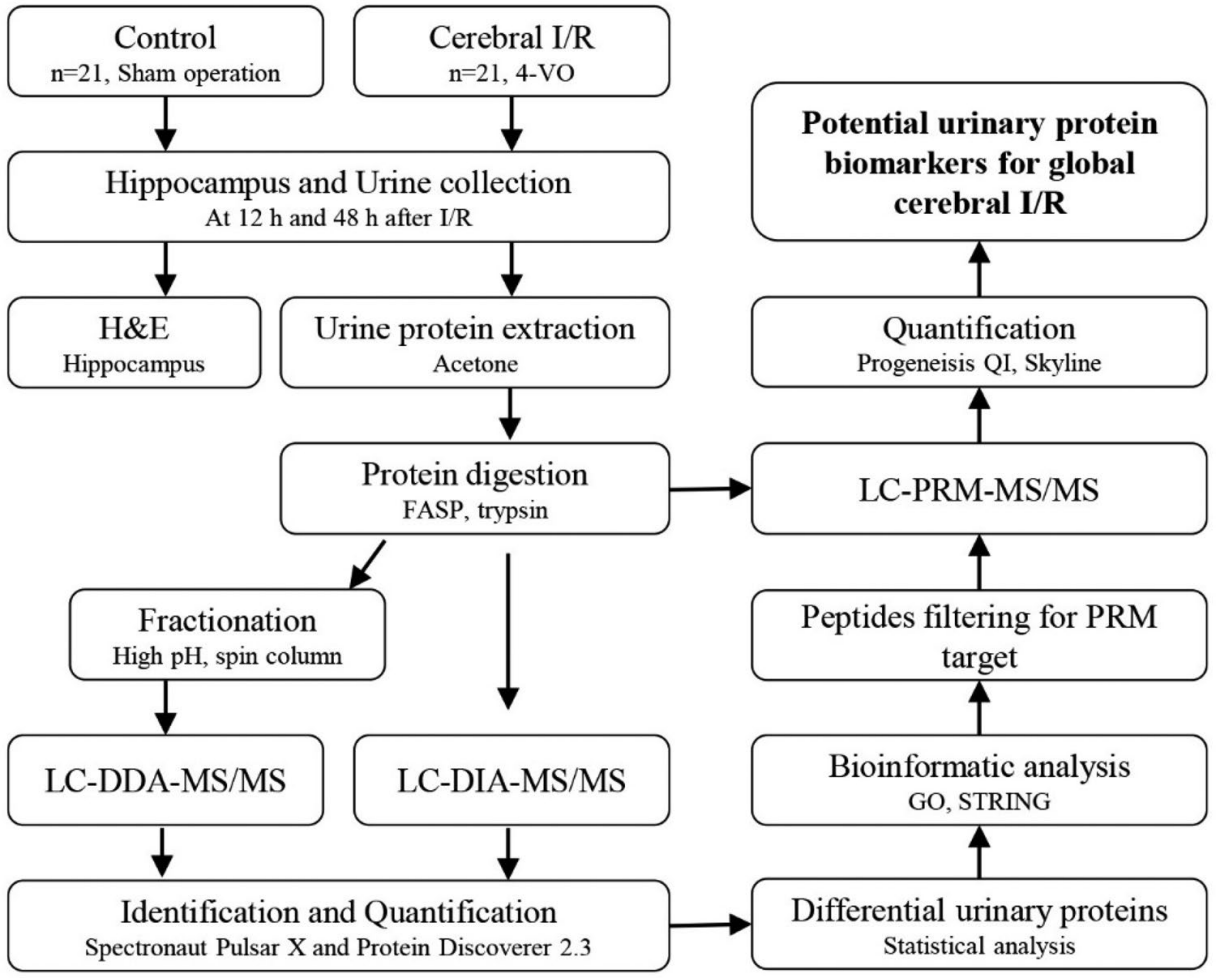
Workflow of this study

In this study, the proteomics technique of data-independent acquisition (DIA) was used to profile the urinary proteome in a global cerebral I/R rat model, and then the altered proteins were validated using the parallel reaction monitoring (PRM) strategy. A summary of the overall experimental approach is presented in Fig. 1. This study aims to explore urinary protein biomarkers of global cerebral I/R injury and provide clues to further understand its molecular biological mechanisms.

## Methods

### Animals

Male Wistar rats (8 weeks, 280–320 g) were purchased from Charles River China (Beijing, China). All animals were maintained on a standard laboratory diet with a controlled indoor temperature (21 ± 2℃), humidity (65–70%), and 12/12-h light–dark cycle conditions. The animal experiments were reviewed and approved by the Qingdao University Hospital Medical Ethics Committee. All methods were carried out in accordance with relevant guidelines and regulations of the National Health Commission and the Ministry of Science and Technology and conformed to the guidelines for animal research.

The Wistar rats were randomly divided into two groups: a sham group (*n* = 21) and an I/R group (*n* = 21). Ischemia was induced by Pulsinelli’s four-vessel occlusion method (Pulsinelli and Buchan 1988). Briefly, rats were fasted for 12 h with water supplied. The next day, the rats were weighed and then anesthetized with an intraperitoneal injection of 1% pentobarbital sodium (40 mg/kg body weight). For rats in the I/R group, firstly, the bilateral pterygoid foramina were surgically exposed and the bilateral vertebral arteries were coagulated; secondly, the bilateral common carotid arteries were surgically exposed and clamped shut with artery clamps for 10 min. For rats in the sham group, vessels were exposed but without occlusion.

The experiment was conducted in two phases; for details, see Fig. 1. For the discovery phase, differentially expressed urinary proteins were identified by label-free DIA quantification in twenty-one independent samples from the control group (7 samples) and the I/R group at 12 and 48 h (7 samples per time point). For the validation phase, the 21 remaining urine samples (7 from the control group and 7 per time point from the I/R group at 12 and 48 h) were evaluated by targeted quantification with PRM.

### Histological Analysis

For histopathology, three rats in the I/R group and three rats in the control group were randomly sacrificed at 12 h and 48 h after I/R. The hippocampus was harvested and then quickly fixed in 10% neutral buffered formalin. The formalin-fixed tissues were embedded in paraffin, and then sectioned (4 μm) and stained with hematoxylin and eosin (H&E) to reveal histopathological lesions.

### Urine Collection and Sample Preparation

Urine samples were collected from the control and I/R groups at 12 and 48 h after I/R. Rats were individually placed in metabolic cages for 4 h. During urine collection, food was withheld from the rats to prevent the urine samples from being contaminated. After collection, the urine samples were immediately centrifuged at 2000* g* for 30 min at 4 °C and then stored at −80 °C.

#### Urinary Protein Extraction

Urine samples were centrifuged at 12,000* g* for 30 min at 4 °C. Five volumes of prechilled acetone were added after the pellets were removed, and mixed samples were precipitated at 4 °C overnight. Then, lysis buffer (8 mol/L urea, 2 mol/L thiourea, 50 mmol/L Tris, and 25 mmol/L DTT) was used to dissolve the pellets. The Bradford protein assay was used to determine the protein concentration of each sample.

#### Tryptic Digestion

Trypsin (Promega, USA) was used, via filter-aided sample preparation methods (Wisniewski et al. 2009). Briefly, 50 µg of the protein sample was loaded onto a 10-kDa filter unit (Pall, USA). DTT (4.5 mM) was added to the protein solution for 1 h at 37 °C. Indoleacetic acid was added (10 mM) for 30 min at room temperature in the dark. The proteins were digested with trypsin (enzyme-to-protein ratio of 1:50) for 16 h at 37 °C. After desalting on Oasis HLB cartridges (Waters, USA), the peptide samples were lyophilized for trap column fractionation and LC–MS/MS analysis.

### Spin Column Separation

Pooled peptide samples were fractionated via a high-pH reversed-phase peptide fractionation kit (Thermo Pierce, USA) according to the manufacturer’s instructions. Briefly, 50 µg of a pooled peptide sample was fractioned. A step gradient of increasing acetonitrile concentrations (5, 7.5, 10, 12.5, 15, 17.5, 20, and 50% acetonitrile) was applied to elute the bound peptides. Ten different fractions were collected by centrifugation, including the flow-through fraction. The fractionated samples were dried and then resuspended in 20 μL of 0.1% formic acid. Three microliters of each sample was analyzed by LC–MS/MS.

### LC–MS/MS Setup for DDA and DIA

An Orbitrap Fusion Lumos Tribrid mass spectrometer coupled with an EASY-nLC 1000 HPLC system (Thermo Scientific, Germany) was used. For retention time stability, the same LC settings were used for DDA-MS and DIA-MS modes. The peptides were loaded onto a trap column (75 µm × 2 cm, 3 µm, C18, 100 A°). Then, it was transferred to a reversed-phase analytical column (50 µm × 250 mm, 2 µm, C18, 100 A°). The eluted gradient was set from 5 to 30% (buffer: 80% acetonitrile 0.1% in formic acid; flow rate of 0.8 μL/min) for 90 min. The calibration kit (iRT kit from Biognosys, Switzerland) reagent was spiked at a concentration of 1:20 v/v in all samples, in order to enable fully automated and sensitive signal processing.

For DDA-MS mode, parameters were set as follows: the full scan was acquired at 60,000 from 350 to 1550 m/*z*, the cycle time was set to top speed mode (3 s), the automatic gain control (AGC) was set to 1E6, and the maximum injection time was set to 50 ms. MS2 scans were acquired with an isolation window of 2 Da at a resolution of 15,000 and higher-energy collisional dissociation (HCD) (collision energy of 32%); the AGC target was set to 5E4 and the maximum injection time was 30 ms.

For the DIA-MS method, parameters were set as follows: the variable isolation window DIA method with 26 windows was developed (Table S1). The full scan was set at a resolution of 60,000 over an *m/z* range of 350 to 1200, followed by DIA scans with a resolution of 30,000, HCD collision energy of 32%, AGC target of 1E6, and maximal injection time of 50 ms.

### LC–MS/MS Setup for PRM

For the PRM-MS method, thirty-two individual samples were analyzed in PRM mode. Ultimately, 255 peptides were scheduled, and the retention time (RT) segment was set to 8 min for each targeted peptide (Table S2). The normalized collision energy was fixed at 30%, and the quadrupole isolation window was fixed at 1.6 Da. The other parameters were the same as described in the last paragraph.

### Label-Free DIA Quantification Analysis

To generate the spectral library, the raw data files acquired for the ten fractions in DDA mode were processed using Proteome Discoverer (version 2.3; Thermo Scientific, Germany) with SEQUEST HT against the SwissProt *Rattus* database (released in May 2019, containing 8086 sequences) appended with the iRT peptide sequences. The search parameters were set as follows: parent ion mass tolerance of 10 ppm; fragment ion mass tolerance of 0.02 Da; fixed modification of carbamidomethylated cysteine (+ 58.00 Da); and variable modifications of oxidized methionine (+ 15.995 Da) and deamidated glutamine and asparagine (+ 0.984 Da). For other settings, the default parameters were used. A false discovery rate (FDR) cutoff of 0.01 was applied at the protein level. The results were then imported to Spectronaut™ Pulsar (Biognosys, Switzerland) software to generate the spectral library (Bruderer et al. 2015).

The raw DIA-MS files were imported into Spectronaut Pulsar with the default settings. In detail, a non-linear iRT calibration strategy and a dynamic window for the XIC extraction window were used. Local mass calibration and cross-run normalization were enabled to correct for systematic variance in LC–MS performance (Callister et al. 2006). As implemented in Spectronaut Pulsar Protein, the inference was performed on the principle of parsimony using the ID picker algorithm (Zhang et al. 2007). *Q* value cutoff of 0.01 (corresponding to an FDR of 1%) was used as a filter for all results. Peak areas of the respective fragment ions for MS2 were calculated as peptide intensity.

### PRM-MS Quantification Analysis

Skyline (version 3.6.1 10,279) (MacLean et al. 2010) was used to build the spectrum library and filter peptides for PRM analysis. For each protein, 2–6 associated peptides were selected using the following rules: (1) identification in the untargeted analysis with a *q* value < 1%, (2) complete digestion by trypsin, (3) containing 8–18 amino acid residues, (4) fixed carbamidomethylation of cysteine, and (5) exclusion of the first 25 amino acids at the N-terminus of proteins. Finally, forty-four proteins with 255 peptides were scheduled for PRM analysis. Based on the pooled sample analysis, the RT segment was set to 8 min for each targeted peptide with its expected RT in the center.

All of the PRM-MS data were processed with Skyline. The RT location and integration boundaries were manually adjusted to exclude interfering regions. The summation of intensities from its corresponding transitions was calculated as each protein’s intensity. The transition settings were as follows: ion charge + 1; precursor charges + 2, + 3; ion type b, y, p; product ions from ion 3 to last ion −1; ion match tolerance 0.02 m/*z*; select the 6 most intense product ions. Normalization was performed according to the summed intensity.

### Bioinformatics Analysis

The Database for Annotation, Visualization and Integrated Discovery (DAVID) 6.8 (https://david.ncifcrf.gov/) was used to perform the functional annotation of the differential urinary proteins identified at 12 and 48 h after I/R injury. In this study, significant GO enrichment was defined as *p* < 0.05. Protein–protein interaction networks were constructed via the STRING database (http://www.string-db.org), which is a database of known and predicted protein interactions, including direct (physical) and indirect (functional) associations.

The differential proteins were selected using one-way ANOVA, and *p*-values were adjusted by the Benjamini and Hochberg method. Significance was defined by a *p*-value of < 0.05 and a fold change of 1.5.

## Results and Discussion

### Histopathological Damage in the Hippocampus

**Fig. 2:**
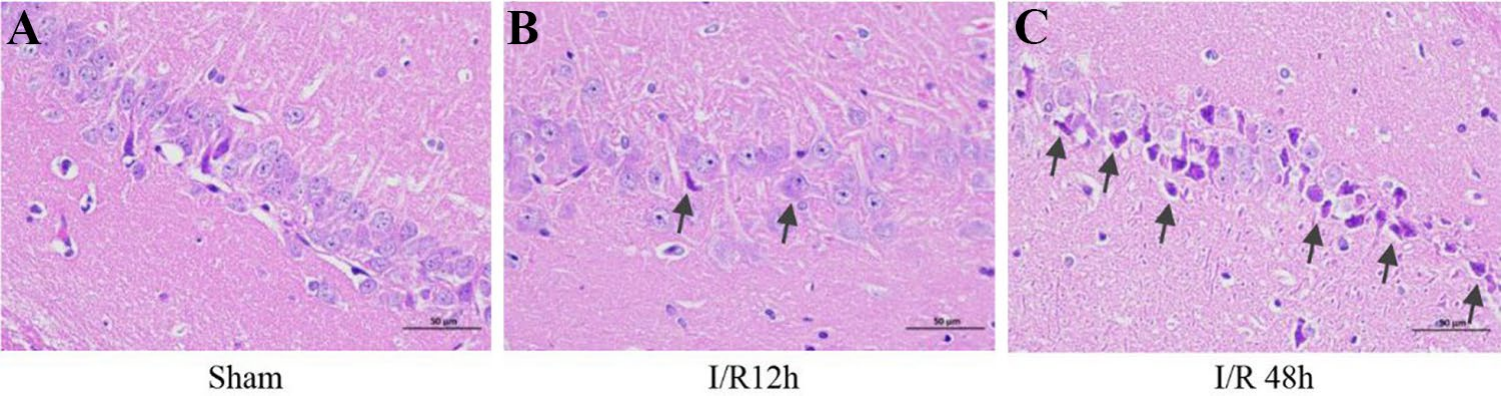
HE staining assessment of neuronal morphology in the hippocampal CA1 area. **A** The sham group showed an orderly arrangement of neurons with complete cell structure; **B** the I/R 12 h group showed a reduced number of neurons, but their structure was complete, and there were no typical apoptotic cells; **C** the I/R 48 h group showed a disorderly arrangement of cells, and the cytoplasm and nucleoli were stained deeply, indicating nuclear pyknosis. Scale bars = 50 µm

To evaluate ischemia and histological damage, H&E staining was conducted on brain sections of the hippocampal CA1 region, which is most well known as being selectively vulnerable following ischemia. HE staining indicated that no neuron morphology abnormalities were observed in the sham group (Fig. 2A). At reperfusion for 12 h, the number of neurons was reduced, the structure was complete, and no typical apoptotic cells were observed (Fig. 2B). With the reperfusion time prolonged to 48 h, the number of intact neurons decreased significantly, besides the shrunken cell bodies and nuclear pyknosis were observed (Fig. 2C).

### Urine Proteome Changes

To preliminarily investigate how the urine proteome changes with I/R progression, twenty-one urine samples from the sham and I/R group (12 h and 48 h) were analyzed via a label-free DIA workflow.

To generate spectral library A, fractions separated with a spin column were analyzed by DDA-MS and then processed using Proteome Discoverer (version 2.3) and Spectronaut Pulsar X. The library included eight DDA analyses of fractions resulting in 1152 protein groups and 6260 peptides with at least one unique peptide and a *Q* value < 0.01. Raw DIA-MS data files acquired with 30 refined isolation windows from the twenty-one individual urine samples were loaded into Spectronaut Pulsar X. Overall, a total of 866 (699 on average) protein groups were identified from twenty-one biological replicates. All identification and quantitation details are listed in supporting Table S3.

One hundred and sixty-four proteins significantly differed in the urine samples compared to the control samples (1.5-fold change, *p* < 0.05). There were 59 and 123 altered urinary proteins at 12 and 48 h, respectively, after I/R (Table S4–5). Eighteen proteins changed significantly at both 12 and 48 h (Table 1). Among these proteins, 7 showed an overall upregulated or downregulated trend: T-kininogen 2, prostaglandin-H2 D-isomerase, 14–3-3 protein theta, Ig gamma-2B chain C region, cathepsin Z, parvalbumin alpha, and hepcidin. This may suggest that these proteins have the potential to be used for the early detection of cerebral I/R injury.

**Table 1:** The urinary proteins that were consistently altered at 12 and 48 h after I/R injury

UniProt ID	Protein name	12 h		48 h	
		FC	*p*-value	FC	*p*-value
P62260	14–3-3 protein epsilon	2.9	4.5E − 02	2.3	2.1E − 02
D3ZTV3	Leucine-rich repeat transmembrane protein FLRT2	2.8	3.1E − 02	2.3	2.7E − 03
Q6P9T8	Tubulin beta-4B chain	2.8	3.3E − 02	2.3	6.8E − 03
Q5ZQU0	Sushi, nidogen and EGF-like domain-containing protein 1	2.3	3.8E − 02	2.3	1.9E − 02
P08932	T-Kininogen 2	1.6	3.6E − 02	2.3	3.2E − 03
P22057	Prostaglandin-H2 D-isomerase	− 1.5	4.4E − 02	− 2.1	8.5E − 03
Q9EQX6	Platelet-derived growth factor C	− 1.6	3.8E − 02	− 1.6	1.5E − 02
O89117	Beta-defensin 1	− 1.6	3.0E − 02	− 1.5	3.4E − 02
Q9WVH8	Fibulin-5	− 1.7	8.1E − 03	− 1.7	2.2E − 02
P68255	14–3-3 protein theta	− 1.7	2.0E − 02	− 2.4	4.3E − 03
P14046	Alpha-1-inhibitor 3	− 2.1	1.6E − 02	− 1.9	2.4E − 02
P20761	Ig gamma-2B chain C region	− 2.2	2.9E − 02	− 2.4	2.1E − 02
P05544	Serine protease inhibitor A3L	− 2.3	5.2E − 03	− 1.6	4.6E − 02
Q9R1T3	Cathepsin Z	− 2.0	9.0E − 03	− 2.2	1.3E − 02
O70534	Protein delta homolog 1	− 2.3	2.6E − 04	− 1.9	3.3E − 03
P02625	Parvalbumin alpha	− 3.2	2.0E − 02	− 4.6	1.3E − 02
Q99MH3	Hepcidin	− 3.4	1.1E − 03	− 4.7	1.6E − 02
P17559	Uteroglobin	− 4.7	4.6E − 02	− 3.1	3.9E − 02

“ − ” means a downward trend

### Function Annotation of Differentially Expressed Proteins

**Fig. 3:**
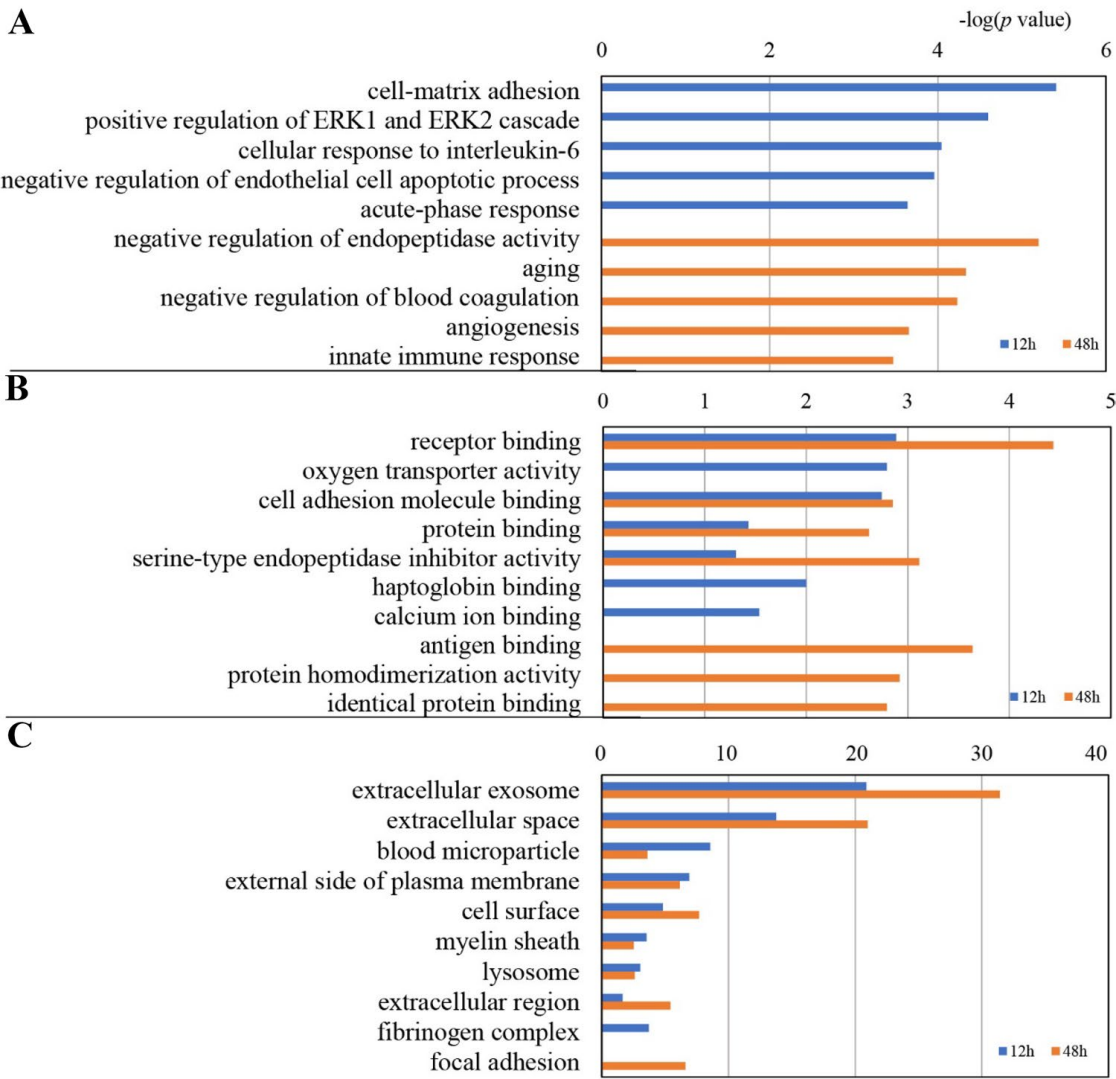
Functional analysis of differentially expressed proteins at 12 h and 48 h after I/R. **A** Biological process category; **B** molecular function category; **C** cellular component category

The functional annotation of differentially expressed proteins at 12 and 48 h consisted of sorting them into the “biological process,” “cellular component,” and “molecular function” categories using DAVID (Fig. 3). One hundred and sixty-four differentially expressed proteins were annotated. In the biological process category, cell–matrix adhesion, positive regulation of ERK1 and ERK2 cascade, cellular response to interleukin-6, negative regulation of endothelial cell apoptotic process, and acute-phase response were overrepresented at 12 h after 12-h I/R. At 48 h after I/R, negative regulation of endopeptidase activity, aging, negative regulation of blood coagulation, angiogenesis, and innate immune response were overrepresented (Fig. 3A).

In the molecular function category, receptor binding, serine-type endopeptidase inhibitor activity, cell adhesion molecule binding, and protein binding were overrepresented at both time points. Oxygen transporter activity was overrepresented at 12 h after I/R (Fig. 3B). In the cellular component category, most of these differentially expressed proteins were associated with extracellular exosomes, extracellular space, blood microparticles, and the external side of the plasma membrane (Fig. 3C).

### Protein–Protein Interactions of Differentially Expressed Proteins

**Fig. 4:**
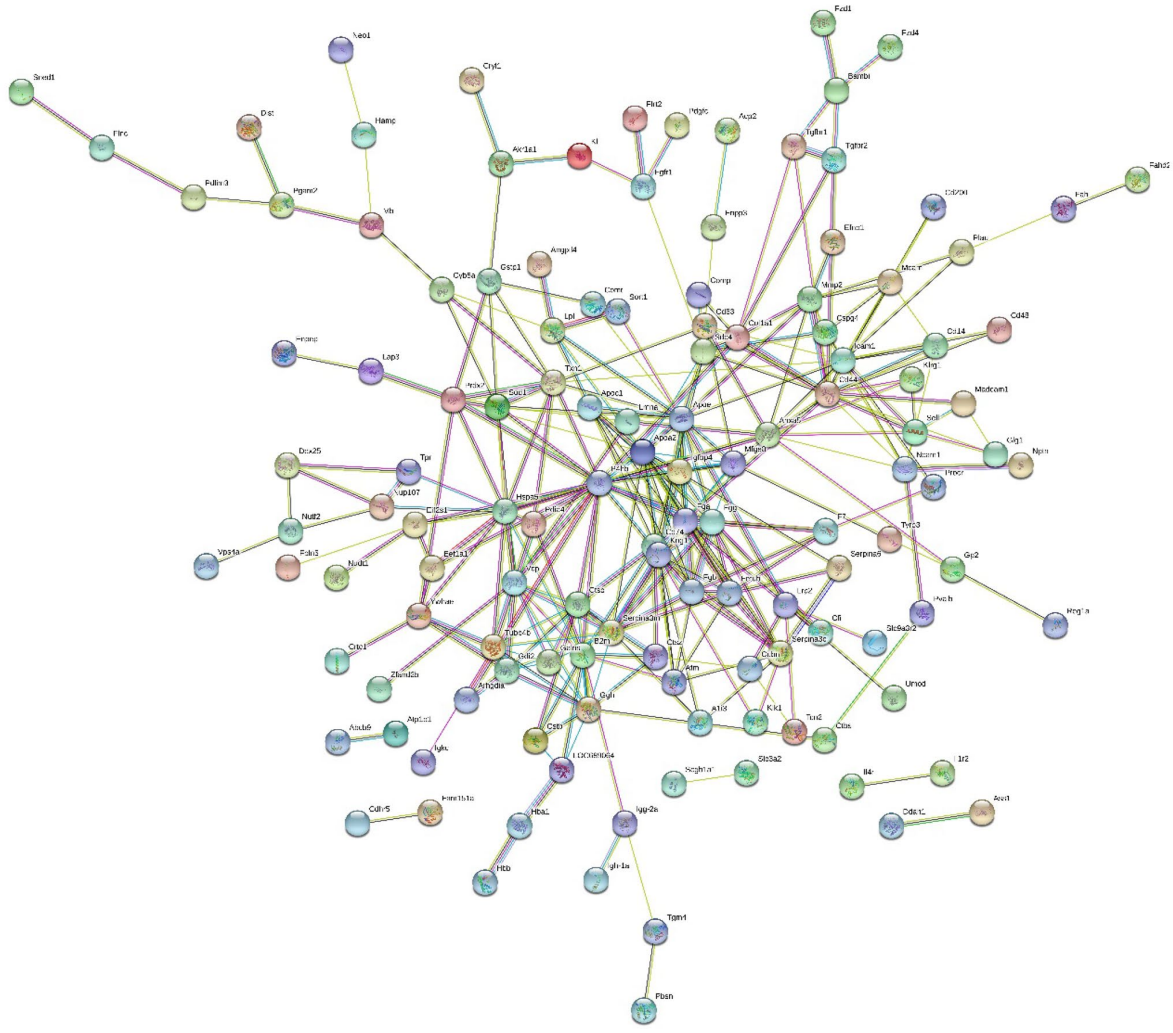
STRING PPI network analysis of the 164 differentially expressed proteins in I/R rats. The average node degree was 3.73, the average local clustering coefficient was 0.466, and the PPI enrichment *p*-value was less than 1.0E − 16

To better understand the pathogenic mechanisms in global cerebral I/R, a protein–protein interaction (PPI) network for 164 changed proteins was constructed using STRING (Fig. 4). The STRING PPI network analysis showed that the average node degree was 3.73, the average local clustering coefficient was 0.466, and the PPI enrichment *p*-value was less than 1.0E − 16. The above results revealed that these proteins had more interactions among themselves than would be expected for a random set of proteins of similar size. Such an enrichment pattern indicates that the proteins are at least partially biologically connected as a group in I/R.

### PRM Validation and Analysis of Differentially Expressed Proteins

In the validation phase, 255 peptides corresponding to seventy-one proteins were finally scheduled for PRM-MS analysis in another 21 urine samples. Overall, fifteen proteins having human orthologs were significantly altered at multiple time points (1.5-fold change, adjust *p* < 0.05), including 8 increased and 7 decreased proteins (Table 2). The expression trends of the corresponding proteins were consistent with the results from the DIA discovery quantification.

**Table 2:** Differentially expressed urinary proteins validated by PRM analysis

UniProt ID	Human homolog	Protein name	Trend	Related to brain I/R injury
P02680	P02679	Fibrinogen gamma chain	↑	Plasma (Jood et al. 2008; Cheung et al. 2008), brain (Zou et al. 2019)
P35444	P49747	Cartilage oligomeric matrix protein	↓	
Q09030	Q03403	Trefoil factor 2	↓	Plasma (Hijazi et al. 2020)
P10247	P04233	H-2 class II histocompatibility antigen gamma chain	↓	
P08932	P01042	T-Kininogen 1	↑	Blood (Sabater-Lleal et al. 2012)
Q9R1T3	Q9UBR2	Cathepsin Z	↓	Plasma (Hijazi et al. 2020)
P22057	P41222	Prostaglandin-H2 D-isomerase	↓	
P02625	P20472	Parvalbumin alpha	↓	Hippocampus (Chavez-Valdez et al. 2018)
Q99MH3	P81172	Hepcidin	↓	Plasma (Slomka et al. 2015; Petrova et al. 2016), brain (Ding et al. 2011)
P02650	P02649	Apolipoprotein E	↑	Brain (Tukhovskaya et al. 2009), plasma (Khan et al. 2013)
Q5M871	O60667	Fas apoptotic inhibitory molecule 3	↑	
Q08463	Q9UP38	Frizzled-1	↑	Brain (Matei et al. 2018)
P43303	P27930	Interleukin-1 receptor type 2	↑	
P29598	P00749	Urokinase-type plasminogen activator	↑	
P10252	P09326	CD48 antigen	↑	

**Fig. 5:**
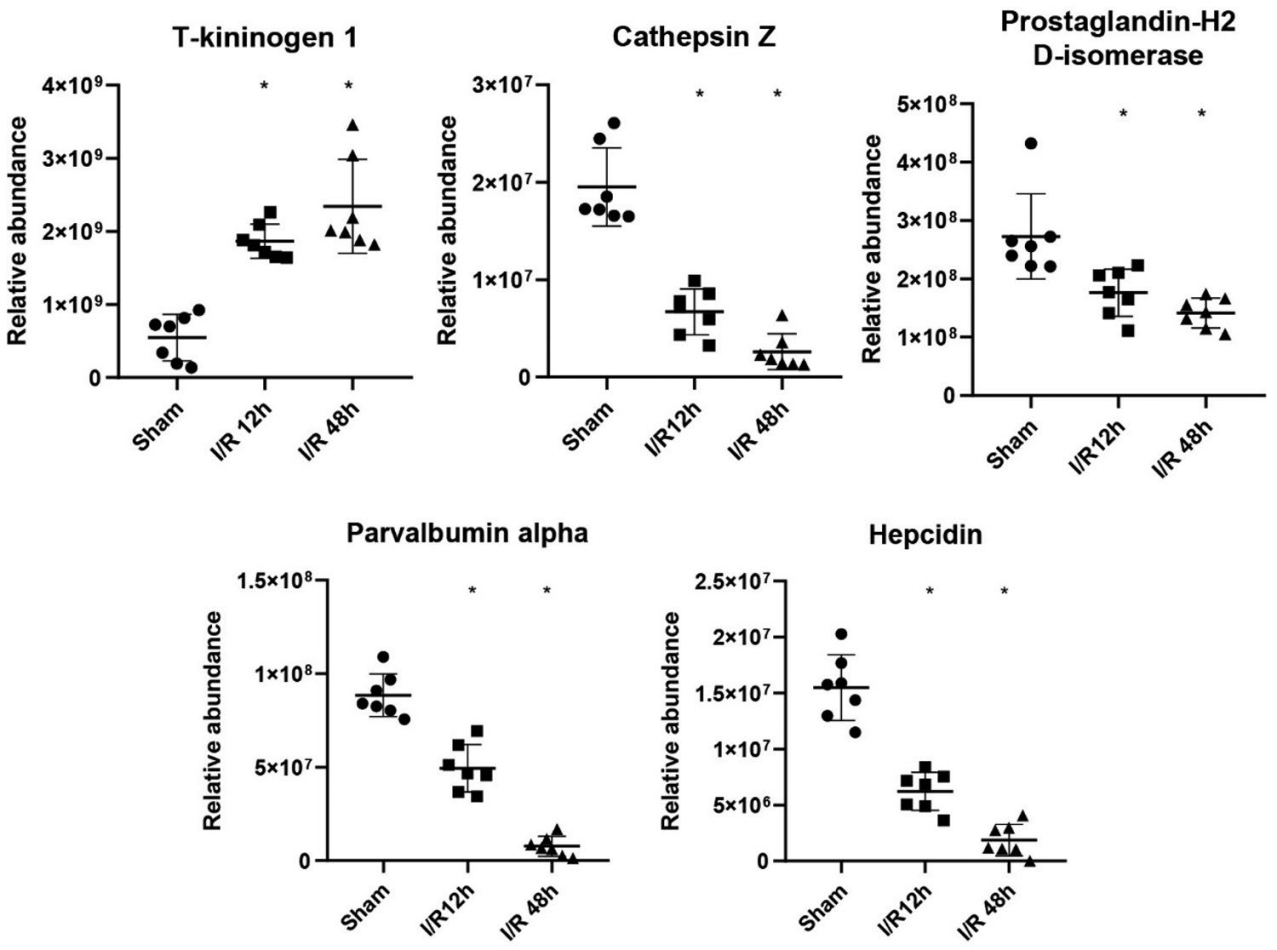
Abundance of candidate urine biomarkers in I/R rats by PRM quantification. The *x*‐axis represents different groups, and the *y*‐axis represents the area of intensity based on PRM quantification. * *p* < 0.05

Nine proteins were changed significantly at 12 h after I/R, when no obvious histopathological changes had yet appeared; these proteins included fibrinogen gamma chain (FGG), cartilage oligomeric matrix protein, trefoil factor 2, H-2 class II histocompatibility antigen gamma chain, T-kininogen 1, cathepsin Z, prostaglandin-H2 D-isomerase, parvalbumin alpha, and hepcidin. These 9 differential proteins may provide important clues for the early screening of cerebral I/R injury. Of these 9 early candidate biomarkers, 5 proteins showed an overall upregulated or downregulated trend at 12 and 48 h after I/R injury, including T-kininogen 1, cathepsin Z, prostaglandin-H2 D-isomerase, parvalbumin alpha, and hepcidin (Fig. 5).

These 5 differential proteins may reflect the progression of cerebral I/R injury, and serve as potential biomarkers for prognostic evaluation of cerebral I/R injury. Of these 9 candidate biomarkers, 6 proteins were previously reported to be closely associated with cerebral I/R injury. Plasma fibrinogen was independently associated with overall ischemic stroke and all subtypes, both in the acute stage (*p* < 0.001) and at the 3-month follow-up (*p* < 0.05) (Jood et al. 2008). In a case–control study, FGG was associated with a reduced risk of ischemic stroke (Cheung et al. 2008). FGG was also highly expressed in the ischemic penumbra of focal cerebral ischemia rats (Zou et al. 2019). Trefoil factor 2 was upregulated in the plasma of atrial fibrillation patients with ischemic stroke (Hijazi et al. 2020). The mRNA expression of T-kininogen 1 was upregulated in the blood of idiopathic thrombophilia (Sabater-Lleal et al. 2012). Cathepsin Z was upregulated in the plasma of atrial fibrillation patients with ischemic stroke (Hijazi et al. 2020). Parvalbumin alpha protein and mRNA were reduced in the hippocampal tissue of C57BL6 mice with unilateral right carotid ligation. Plasma/serum hepcidin levels were significantly higher in acute ischemic stroke patients than those in the control group (Slomka et al. 2015; Petrova et al. 2016). The hepcidin mRNA levels and hepcidin/prohepcidin protein levels are upregulated in the ischemic brain (Ding et al. 2011).

The other 6 differential proteins were upregulated only at 48 h after I/R injury, when there was certain histopathological damage in the hippocampus; these proteins included apolipoprotein E, Fas apoptotic inhibitory molecule 3, frizzled-1, interleukin-1 receptor type 2, urokinase-type plasminogen activator, and CD48 antigen. These differentially expressed proteins may indicate the extent of cerebral I/R injury. Two of these differentially expressed proteins were associated with cerebral I/R injury. Apolipoprotein E (APOE) is the primary apolipoprotein synthesized in the brain in response to ischemia–reperfusion injury with known neuroprotective effects exerted through antioxidant, anti-inflammatory, anti-excitotoxic, and neurotrophic mechanisms (Tukhovskaya et al. 2009). The APOE genotype showed a positive dose–response association with ischemic stroke in people of European ancestry (Khan et al. 2013). Frz1 expression was significantly decreased in the brain tissue of middle cerebral artery occlusion rats (Matei et al. 2018).

## Conclusion

In this study, fifteen differential urinary proteins having human orthologs were identified and validated as the potential urinary markers associated with cerebral I/R injury. Eight of the differential proteins were reported to be associated with cerebral I/R injury, namely fibrinogen gamma chain, trefoil factor 2, T-kininogen 1, cathepsin Z, parvalbumin alpha, hepcidin, apolipoprotein E, and Frz1. These findings may provide important clues for the early screening and monitoring of cerebral I/R injury and further understanding of its molecular biology mechanisms.

## Supplementary Information

Below is the link to the electronic supplementary material.

Supplementary file1 (XLSX 233 KB)

## Data Availability

The datasets used and/or analyzed during the current study are available from the corresponding author on reasonable request.
